# Avenanthramide C from germinated oats exhibits anti-allergic inflammatory effects in mast cells

**DOI:** 10.1038/s41598-019-43412-2

**Published:** 2019-05-03

**Authors:** Hima Dhakal, Eun-Ju Yang, Soyoung Lee, Min-Jong Kim, Moon-Chang Baek, Byungheon Lee, Pil-Hoon Park, Taeg Kyu Kwon, Dongwoo Khang, Kyung-Sik Song, Sang-Hyun Kim

**Affiliations:** 1 Cell & Matrix Research Institute, School of Medicine, Kyungpook National University, Daegu, Republic of Korea; 2 Department of Pharmacology, School of Medicine, Kyungpook National University, Daegu, Republic of Korea; 3 Research Institute of Pharmaceutical Sciences, College of Pharmacy, Kyungpook National University, Daegu, Republic of Korea; 4 Immunoregulatory Materials Research Center, Korea Research Institute of Bioscience and Biotechnology, Jeongeup, Republic of Korea; 5 Department of Molecular Medicine, School of Medicine, Kyungpook National University, Daegu, Republic of Korea; 6 Department of Biochemistry and Cell Biology, School of Medicine, Kyungpook National University, Daegu, Republic of Korea; 7 College of Pharmacy, Yeungnam University, Gyeongsan, Republic of Korea; 8 Department of Immunology, School of Medicine, Keimyung University, Daegu, Republic of Korea; 9 Department of Physiology, School of Medicine, Gachon University, Incheon, Republic of Korea; 10 GHAM BioPharm Co. Ltd., College of Pharmacy, Kyungpook National University, Daegu, Republic of Korea

**Keywords:** Acute inflammation, Drug development

## Abstract

Mast cells play a crucial role in allergic diseases *via* the release of inflammatory mediators, particularly histamine and pro-inflammatory cytokines. Avenanthramide (Avn) C, a polyphenol found mainly in oats, is known to exhibit various biological properties. In this study, we aimed to evaluate the effectiveness of Avn C from germinated oats against mast cell-mediated allergic inflammation. For the *in vitro* study, RBL-2H3, mouse bone marrow-derived mast cells and rat peritoneal mast cells were used. Avn C (1–100 nM) inhibited the immunoglobulin (Ig)E-stimulated mast cells degranulation by suppressing phosphorylation of phosphoinositide 3-kinase and phospholipase Cγ1 and decreasing intracellular calcium levels. It inhibited IgE-stimulated secretion of inflammatory cytokines *via* suppression of FcεRI-mediated signaling proteins Lyn, Syk, Akt, and nuclear factor-κB. To verify the effects of Avn C *in vivo*, ovalbumin-induced active systemic anaphylaxis (ASA) and IgE-mediated passive cutaneous anaphylaxis (PCA) models were used. Oral administration of Avn C dose-dependently attenuated the ASA reactions, as evidenced by the inhibition of hypothermia and reduction of elevated serum histamine, IgE, and interleukin-4 levels. Avn C also inhibited the PCA reactions, such as ear swelling and plasma extravasation. Our results suggested that Avn C from germinated oats might be a possible therapeutic candidate for mast cell-mediated allergic inflammation.

## Introduction

Allergic diseases are very common with continuously increasing prevalence, where they affect 20% of the world’s population^1^. The increasing prevalence of allergic disease is linked to the presence of environmental allergens, which are innocuous to human health^2^. Mast cells are recognised as effector cells, which play an important role in allergic disease via secretion of inflammatory mediators^3^. These cells are present in vascularised tissues, particularly in the body part exposed to external environment, including gastrointestinal tract and airway epithelium^2^. Crosslinking of immunoglobulin (Ig)E with its high-affinity receptor, FcεRI activates the mast cells^4^. Activated mast cells release a large amount of biologically active mediators, such as histamine, pro-inflammatory cytokines, chemokines, lipid-derived mediators, and angiogenic factors, resulting the allergic inflammatory responses^4^. Among those mediators, histamine is a key player of allergic response, where it leads to vasodilatation and hypothermia, increases the vascular permeability, and triggers the activation of inflammatory cascades^4^.

In mast cells, calcium is essential for degranulation and secretion of inflammatory cytokines^5^. The interaction of IgE and FcεRI initiates phosphorylation of Src family kinases Lyn, Syk, and Fyn and phospholipase C (PLC)γ^6^. Phosphorylation of PLCγ results in production of inositol 1,4,5-triphosphate (IP_3_) and diacylglycerol (DAG) as secondary messengers, which leads to the release of intracellular calcium^5,6^. These are followed by activation of protein kinase C (PKC) and nuclear factor (NF)-κB, leading to degranulation of mast cells and secretion of inflammatory cytokines^7^. The transcription factor, NF-κB regulates the expression of cytokines including tumour necrosis factor (TNF)-α, interleukin (IL)-4, IL-8, IL-6, and IL-13, through proteolytic degradation of IκBα^8,9^.

Oats (*Avena sativa* L.) (Poaceae) has been traditionally used as a remedy for different dermatological disease, such as atopic dermatitis, dry skin, contact dermatitis and psoriasis^10–13^. Protein-free oat plantlet extract and oligomer extract inhibited atopic dermatitis in patients and vasoactive intestinal peptide-induced skin inflammation in human, respectively^11,13,14^. Avenanthramides (Avns) are conjugates of a phenylpropanoid and 5-hydroxy anthranilic acid, which are soluble phenolic compounds, extracted from oats^13^. More than 20 isoforms of Avns have been identified in oat, which vary in the substituents of the cinnamic acid and anthranilic acid rings^15^. Three major isoforms of Avns, Avn A, B, and C have been extensively used^16^. These three major isoforms have shown the anti-oxidant, anti-proliferative, anti-histamine, and anti-inflammatory functions in coronary heart disease, colon cancer, skin inflammation, and skeletal muscles^10,13,17–20^. In addition, the synthetic analogue, dihydro Avn D inhibited substance P-induced mast cell degranulation and calcium release^21^.

Avn C content in oat seed is two-fold higher than that of Avn A or B^22^. Avn C showed the high bioactivity and anti-oxidant effects by inhibiting the growth of colon cancer cells and preventing DNA damage^13,23,24^. In addition, it decreased the viability of tumour cells by activating apoptosis in breast cancer^25^. Furthermore, Avn C and its methylated derivative inhibited the expression of pro-inflammatory cytokines through suppression of NF-κB activation in endothelial cells^20^. A recent protein-ligand docking and molecular dynamics simulation study suggested that Avn C potently inhibits NF-κB-mediated inflammatory response by decreasing IKKβ’s activity in skeletal muscle cells^18^.

In this study, we isolated Avn C from germinated oats. Germination is an important method to improve the properties and content of Avn C in oats^26^. Our study aimed to investigate the anti-allergic inflammatory properties of Avn C isolated from germinated oats on mast cells.

## Results

### Effects of Avn C on mast cell degranulation

**Figure 1:**
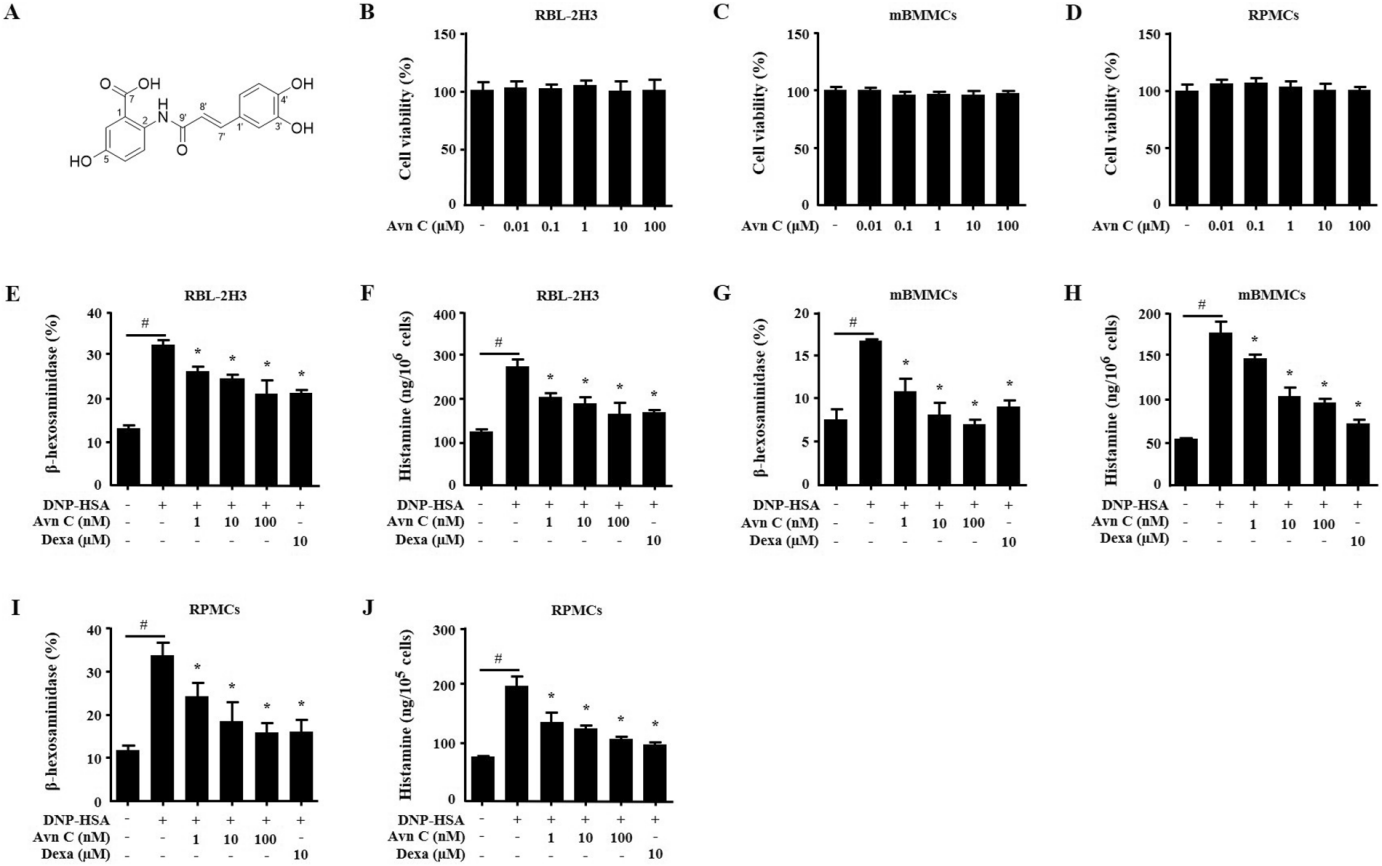
Effects of Avn C on mast cell degranulation. (**A**) Chemical structure of Avn C. (**B**–**D**) RBL-2H3, mBMMCs and RPMCs (3 × 10^4^ cells/well) were pre-treated with or without Avn C, then incubated with MTT. The absorbance was detected using a spectrophotometer. For mast cell degranulation, RBL-2H3 and mBMMCs (5 × 10^5^ cells/well), and RPMCs (3 × 10^4^ cells/well) were sensitised with anti-DNP IgE (50 ng/mL). After incubation overnight, the cells were pre-treated with or without Avn C or Dexa for 1 h and then challenged with DNP-HSA (100 ng/mL). (**E**,**G**,**I**) The level of β-hexosaminidase was measured using β-hexosaminidase substrate buffer. (**F**,**H**,**J**) Histamine level was assayed using the *o*-phthaldialdehyde spectrofluorometric method. Each data presented as a graph represents the means ± SEM of three independent experiments. ^#^Significantly different form the control group at *p* < 0.05. *Significantly different from the DNP-HSA-challenged group at *p* < 0.05. Dexa: dexamethasone.

The chemical structure of Avn C was displayed in Fig. 1A. The possible cytotoxicity of Avn C was first tested using MTT assay. Avn C (0.01–100 µM) treated RBL-2H3, mBMMCs, and RPMCs were incubated for 12 h. Avn C did not show any cytotoxicity up to 100 µM (Fig. 1B–D). Next, we evaluated the effects of Avn C on degranulation of mast cells based on β-hexosaminidase and histamine release. Dexamethasone (Dexa) was used as a positive control drug. IgE/Ag-sensitized RBL-2H3 cells, mouse bone marrow derived mast cells (mBMMCs), and rat peritoneal mast cells (RPMCs) were challenged with dinitrophenyl-human serum albumin (DNP-HSA). Pre-treatment with Avn C (1–100 nM) considerably reduced the β-hexosaminidase and histamine release in a concentration-dependent manner in RBL-2H3 cells (Fig. 1E,F), mBMMCs (Fig. 1G,H), and RPMCs (Fig. 1I,J), compared with that in DNP-HSA challenged cells.

### Effects of Avn C on intracellular calcium levels in mast cells

**Figure 2:**
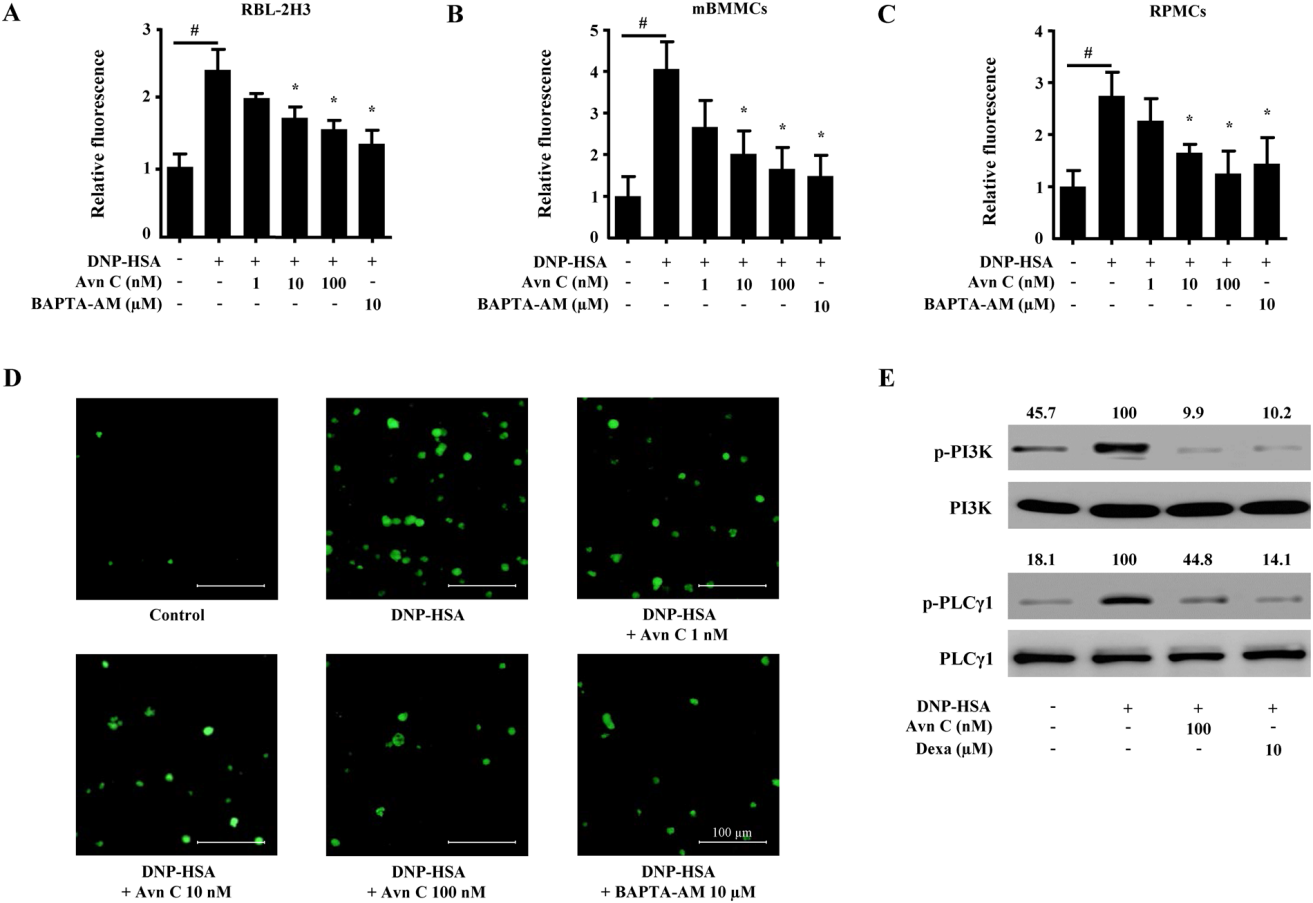
Effects of Avn C on intracellular calcium levels in mast cells. RBL-2H3, mBMMCs, and RPMCs (3 × 10^4^ cells/well) were pre-incubated with Fluo/3AM. Intracellular calcium was detected using a fluorescence plate reader. (**A**–**C**) Intracellular calcium levels in RBL-2H3, mBMMCs, and RPMCs. Each data presented as a graph represents the means ± SEM of three independent experiments. (**D**) Representative fluorescence imaging of intracellular calcium levels in RBL-2H3 cells (original magnification × 200). Fluorescence images represent the 5 random cell sites. Scale bar: 100 μm. (E) RBL-2H3 (1.5 × 10^6^ cells/well) were sensitised with anti-DNP IgE (50 ng/mL). After incubation overnight, the cells were pre-treated with or without Avn C or Dexa for 1 h and then challenged with DNP-HSA (100 ng/mL). The activation of signalling proteins (p-PI3K and p-PLCγ1) was assayed by Western blot analysis (p-: phosphorylated). Cropped blots are shown. Full-length blots are presented in Supplementary Fig. S2. The bands of the total form were used as loading controls. The bands shown are representative of three independent experiments. The Western band intensity was quantified using Image J software. The representative band intensity was digitized to the relative intensity. ^#^Significantly different form the control group at *p* < 0.05. *Significantly different from the DNP-HSA-challenged group at *p* < 0.05. Dexa: dexamethasone.

Increase of intracellular calcium levels triggers the mast cell degranulation^5^. To evaluate the mechanism, by which Avn C in suppressed mast cell degranulation, we measured the intracellular calcium content. The inhibitory effects of Avn C in intracellular calcium content was analysed using fluorescent indicator Fluo-3/AM. When RBL-2H3 cells, mBMMCs, and RPMCs were stimulated with DNP-HSA, intracellular calcium level was significantly elevated. However, pre-treatment with Avn C (1–100 nM) suppressed the intracellular calcium content in a concentration-dependent manner (Fig. 2A–C). Intracellular calcium level was also determined by fluorescent imaging (Fig. 2D). To further investigate the mechanism responsible for inhibition of mast cell degranulation by Avn C, we evaluated the effects of Avn C on the phosphorylation of PI3K and PLCγ1. Results showed that Avn C pre-treatment inhibited DNP-HSA-stimulated phosphorylation of PI3K and PLCγ1 (Fig. 2E).

### Effects of Avn C on secretion of inflammatory cytokines in mast cells

**Figure 3:**
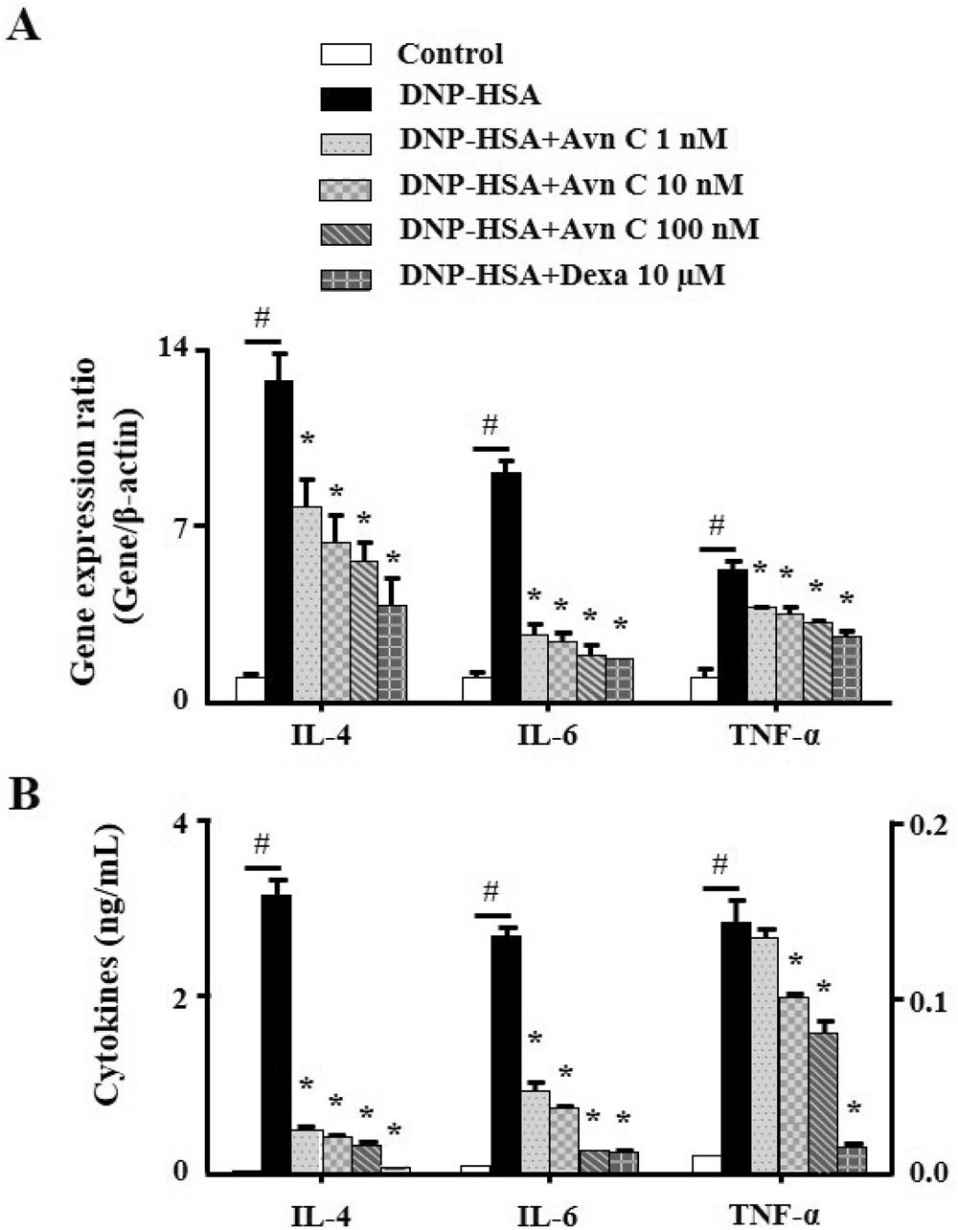
Effects of Avn C on secretion of pro-inflammatory cytokines in mast cells. RBL-2H3 (5 × 10^5^ cells/well) were sensitised with anti-DNP IgE (50 ng/mL). After incubating overnight, the cells were pre-treated with or without drugs Avn C and Dexa for 1 h and then challenged with DNP-HSA (100 ng/mL). (**A**) The gene expression of inflammatory cytokines was determined by qPCR. (**B**) The release of inflammatory cytokines was measured by ELISA. The left Y-axis represents the release of IL-4 and right Y-axis represents the release of IL-6 and TNF-α. Each data presented as a graph represents the means ± SEM of three independent experiments. ^#^Significantly different form the control group at *p* < 0.05. *Significantly different from the DNP-HSA-challenged group at *p* < 0.05. Dexa: dexamethasone.

Cytokines, such as IL-4, IL-6, and TNF-α, released from activated mast cells result in inflammation and promote allergic reactions^8^. To evaluate the effects of Avn C on the expression and release of pro-inflammatory cytokines in RBL-2H3 cells, quantitative polymerase chain reaction (qPCR) and enzyme-linked immunosorbent assay (ELISA) were performed, respectively. Pre-treatment with Avn C (1–100 nM) inhibited the gene expression and release of pro-inflammatory cytokines in a concentration-dependent manner, compared with those in DNP-HSA challenged cells (Fig. 3A,B).

### Effects of Avn C on FcεRI-mediated signalling proteins in mast cells

**Figure 4:**
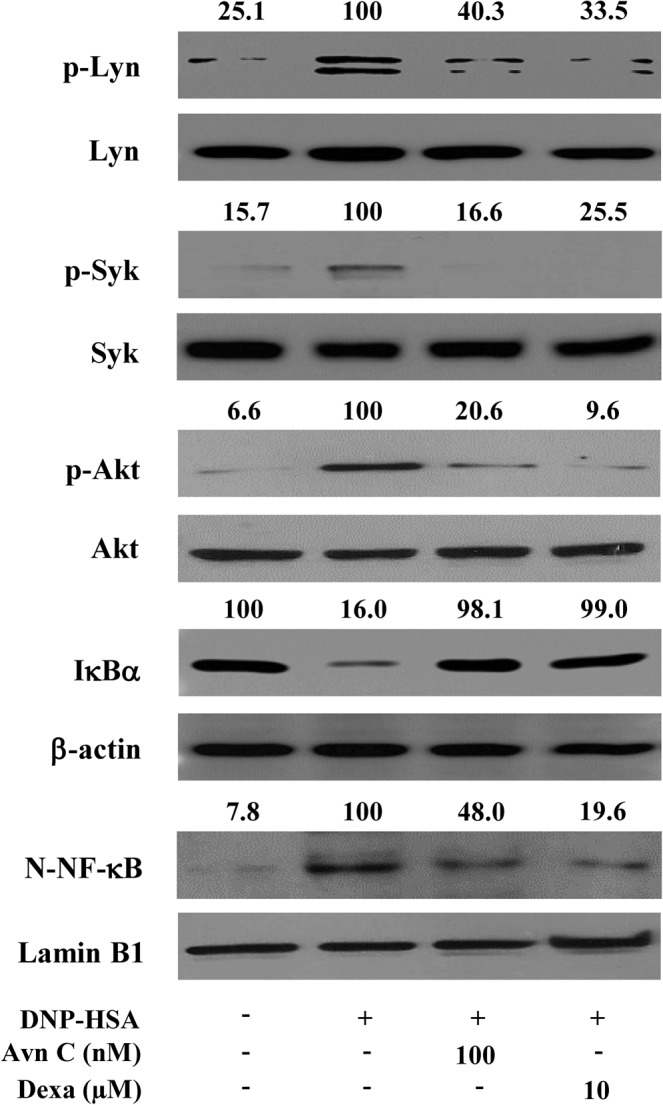
Effects of Avn C on FcεRI-mediated signalling proteins in mast cells. RBL-2H3 (1.5 × 10^6^ cells/well) were sensitised with anti-DNP IgE (50 ng/mL). After incubation overnight, the cells were pre-treated with or without Avn C or Dexa for 1 h and then challenged with DNP-HSA (100 ng/mL). The activation of signalling proteins was assayed by Western blot analysis (N-: nuclear, p-: phosphorylated). Cropped blots are shown. Full length blots are presented in Supplementary Fig. S2. The bands of β-actin, lamin B1, and total form were used as loading controls. The bands shown are representative of three independent experiments. The Western band intensity was quantified by using Image J software. The representative band intensity was digitized to the relative intensity. Dexa: dexamethasone.

Aggregation of FcεRI initiates the activation of Src family kinases, Lyn and Syk. Subsequently, the phosphorylation of Syk activates Akt/NF-κB signalling pathways^7^. Degradation of IκBα and translocation of NF-κB to the nucleus are essential for pro-inflammatory cytokine secretion^7^. To evaluate the mechanism by which Avn C inhibited expression of cytokines in mast cells, we investigated the effects of Avn C on FcεRI-dependent signalling proteins (Lyn, Syk, Akt, and NF-κB). In our findings, Avn C inhibited the phosphorylation of Lyn, Syk, Akt, as well as the degradation of IκBα and nuclear translocation of NF-κB in DNP-HSA challenged RBL-2H3 cells (Fig. 4).

### Effects of Avn C on systemic and local anaphylaxis

**Figure 5:**
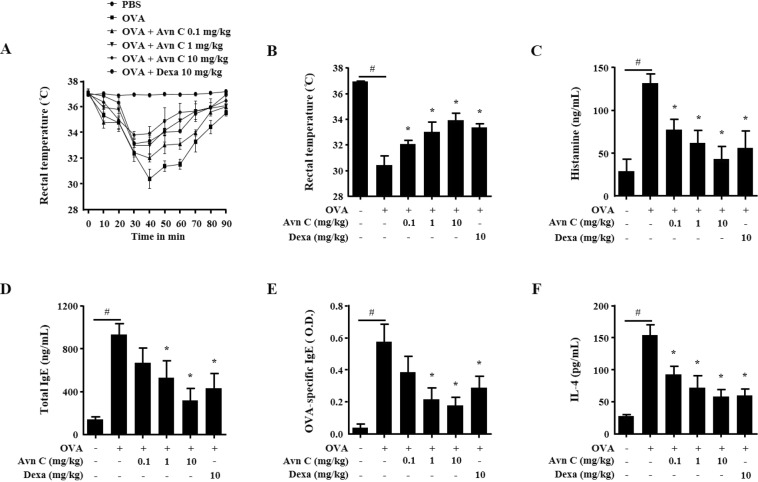
Effects of Avn C on OVA-induced ASA. Each mouse (total *n* = 30, *n* = 5/group) was injected intraperitoneally with OVA mixture (100 μg of OVA and 2 mg of alum adjuvant**)** or PBS on days 0 and 7. Drugs, including Avn C (0.1–10 mg/kg) and Dexa (10 mg/kg), were orally administered on days 9, 11, and 13. On day 14, mice were challenged with an intraperitoneal injection of 200 μg of OVA, and then the rectal temperature was monitored and recorded every 10 min for 90 min. Mice were euthanised after 90 min, and blood samples were collected from the abdominal artery. (**A**) Rectal temperature was measured every 10 min for 90 min. (**B**) Rectal temperature of mice at 30 min. (**C**–**F**) Serum histamine, total IgE, OVA-specific IgE, and IL-4 were detected by ELISA. Each data presented as a graph represents the means ± SEM (*n* = 5/group) of two independent experiments. ^#^Significantly different form the control group at *p* < 0.05. *Significantly different from the DNP-HSA-challenged group at *p* < 0.05. Dexa: dexamethasone.

Ovalbumin (OVA)-induced active systemic anaphylaxis (ASA) model is suitable to evaluate the effectiveness of anti-allergic inflammatory drug candidates^27^. To induce systemic anaphylaxis in mice, a mixture of OVA and alum adjuvant was administered twice, and mice were challenged with OVA. After OVA challenge, the rectal temperatures of the mice were measured for 90 min. During 30–60 min, the body temperature of mouse was decreased, whereas the serum histamine level substantially increased. Oral administration of Avn C (0.1–10 mg/kg), dose-dependently increased the rectal temperature and reduced the serum histamine level (Fig. 5A–C). Additionally, Avn C suppressed the increased levels of serum total IgE, OVA-specific IgE, and IL-4 in OVA challenged mice (Fig. 5D–F).

**Figure 6:**
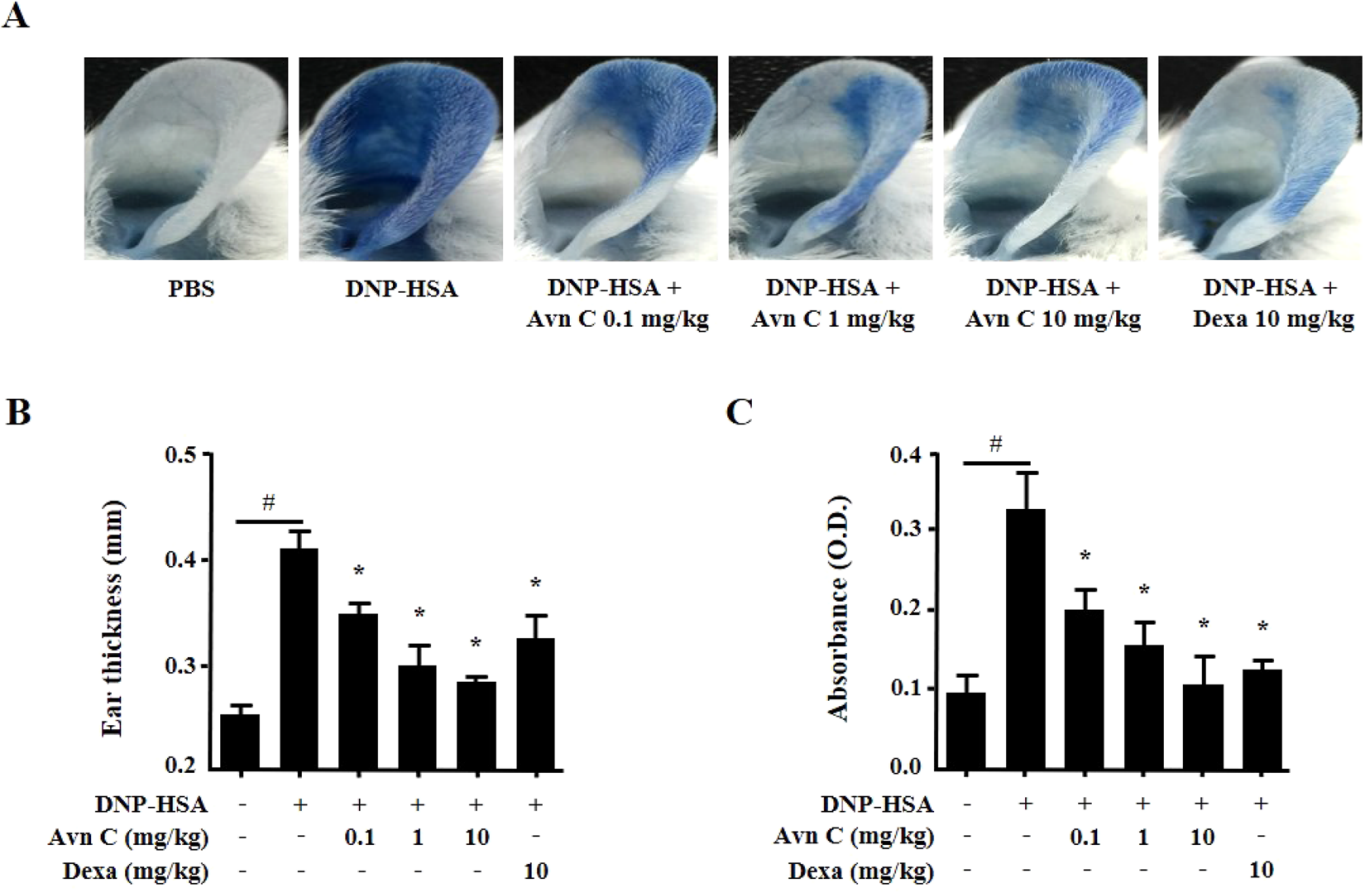
Effects of Avn C on IgE-mediated PCA. The ear skin of mice (total *n* = 30, *n* = 5/group) were sensitised with an intradermal injection of anti-DNP IgE (0.5 μg/site) or PBS for 48 h. Drugs, including Avn C (0.1–10 mg/kg) and Dexa (10 mg/kg) were administered per oral, 1 h before intravenous injection of DNP-HSA (1 mg/mouse) and 4% Evans blue (1:1) mixture. Thirty minutes later, the thickness of both ears was measured, and the ears were collected to measure the pigmentation. The extravasation of dye was detected using a spectrophotometer. (**A**) Representative photographs of ears. (**B**) Thickness of ears. (**C**) Absorbance representing dye extravasations. Each data presented as a graph represents the means ± SEM (*n* = 5/group) of two independent experiments. ^#^Significantly different form the control group at *p* < 0.05. *Significantly different from the DNP-HSA-challenged group at *p* < 0.05. Dexa: dexamethasone.

Passive cutaneous anaphylaxis (PCA) is widely used animal model to demonstrate the local anaphylaxis^28^. Anti-DNP IgE-sensitised mice were challenged intravenously with a mixture of 4% Evans blue and DNP-HSA. The PCA reaction at the site of sensitization was characterized by increased ear swelling and extravasation of Evans blue dye. Oral administered of Avn C (0.1–10 mg/kg) decreased the PCA reaction in a dose-dependent manner (Fig. 6).

## Discussion

Previous studies reported that germinated oats enhance bioavailability of proteins and polyphenol contents by the increase of free amino acid contents^13,29^. Avns are group of naturally occurring polyphenols discovered in oats. Avns have shown potential anti-inflammatory, anti-irritant and anti-oxidant effects^11,13^. The anti-irritant function of oats has also been demonstrated in clinical trials^11^. Compared to other isoforms, Avn C showed higher anti-oxidative, anti-cancerous and anti-inflammatory activities^17,24^. In our study, Avn C, an active component of germinated oats, inhibited mast cell-mediated allergic inflammation.

Mast cells are effector cells in allergic inflammation and life-threatening anaphylaxis^3,4^. Interaction of the allergen-specific IgE with FcεRI on mast cells lead to the release of allergic mediators, such as histamine, proteases, cytokines, and chemokines^8^. Therefore, effective pharmacological modification of mast cell activation could be a proper approach to manage allergic inflammation. Pre-stored mediators, such as β-hexosaminidase and histamine, accumulating in secretory granules of mast cells together with serotonin and proteases are rapidly released after antigen stimulation also known as degranulation^30,31^. Histamine is responsible for inducing allergic reactions, such as vasodilation, hypothermia, oedema, and warmth^31^. In this study, we have done β-hexosaminidase and histamine assay to investigate the mast cell degranulation using various types of mast cells (RBL-2H3, mBMMCs, and RPMCs) and to analyse the anti-allergic inflammatory effects of Avn C. RPMCs and mBMMCs are widely used primary cultured mast cells having the physiological properties of the residing cells *in vivo*. Targeting these cells for drug invention could provide a wide range of therapeutic approaches^32^. In the present study, antigen-stimulated release of β-hexosaminidase and histamine was inhibited by Avn C. Therefore, we suggest that Avn C might exhibit anti-allergic and anti-inflammatory effects by suppressing mast cell degranulation.

Mast cell degranulation is dependent on intracellular calcium levels^33^. Calcium is an important cation, which functions as a secondary messenger in various immune cells^34^. Signalling cascades are initiated by phosphorylation of protein kinases, such as Lyn, Syk, SH2 domain-containing leukocyte protein of 76 kD, Grb2-associated binder 2, degranulation-promoting adaptor protein, and PI3K^35^. These signalling pathways are required for activation of PLCγ, which hydrolyses membrane-bound phosphatidylinositol 4,5-biphosphate into two secondary messengers, IP_3_ and DAG. DAG mediates the activation of PKC, whereas IP_3_ binds to its receptor, IP_3_R, in the endoplasmic reticulum membrane, which triggers the release of calcium from intracellular stores^5,35^. Regulation of intracellular calcium is the major target of effective anti-allergic drugs, as this is necessary for degranulation of mast cells. Our results showed that Avn C treatment suppressed calcium levels in mast cells^5^. Our results suggest that Avn C suppressed the mast cell degranulation by decreasing the intracellular calcium levels.

Increase of intracellular calcium content regulates exocytosis and the expression of inflammatory cytokines in mast cells^5,33^. Mast cells-derived pro-inflammatory cytokines, particularly, IL-4, IL-6, and TNF-α are crucial for the pathogenesis of allergic inflammation^36^. In this study, we showed that Avn C inhibited IgE-induced IL-4, IL-6, and TNF-α expression in mast cells. IL-4 is the major T_H_2 cytokine, which promotes the development antigen-specific IgE through class-switching of B cells^2^. Mast cells express IL-4 in response to IgE stimuli, resulting in allergic inflammation^2^. Conventionally, IL-6 is considered as an inflammatory marker of ongoing inflammation, which promotes the differentiation of CD4^+^ T-cells into T_H_2 cells producing IL-4^37^. Moreover, it has been suggested that IL-6 also contributes to allergic disease through IgE production^38^. TNF-α is a hallmark of inflammatory response, which is secreted by immune cells. TNF-α promotes inflammation, tissue fibrosis, endothelial activation, and increases vascular permeability^6,39^. Therefore, inhibition of pro-inflammatory cytokines released from activated mast cells is the key measure, which is correlated to reduced allergic inflammation.

Expression of pro-inflammatory cytokines is regulated by the transcription factor, NF-κB, which is an important pathogenic factor in acute and chronic inflammation^9^. The role of NF-κB has been extensively investigated in several allergic diseases, where it induces gene expression, leading to the synthesis of inflammatory cytokines^9,31,40^. Furthermore, IgE-induced IL-6 and TNF-α release from mast cells was reduced by various calcium blockers^41^. These effects were mediated by the NF-κB pathway. Intracellular calcium is critical for the proteolytic degradation of IκBα and nuclear translocation of NF-κB^41,42^. In our study, Avn C inhibited the nuclear translocation of NF-κB and release of pro-inflammatory cytokines. These effects were mediated by the suppression of increased intracellular calcium levels. In addition, allergic inflammation mediated by mast cells is linked to the activation of FcεRI-dependent signalling pathway, which phosphorylates Lyn and Syk and induces secretion of inflammatory mediator^43^. In particular, Syk is a crucial tyrosine kinase for the activation of downstream molecules^44^. Phosphorylation of Syk also activates the PI3K/Akt/NF-κB pathway^7^. Moreover, it has been suggested that Akt pathway activates IKK complex, which activates NF-κB^7^. In line with these findings, Avn C significantly suppressed the phosphorylation of Lyn and Syk, which are important intracellular downstream proteins^28^. Therefore, our results suggest that inhibition of FcεRI-proximal signalling pathway led to the suppression of inflammatory cytokines.

As previously described, IgE and mast cells have been convincingly associated with acute allergic reactions, which result from the release of inflammatory mediators^2,8^. To evaluate the potential efficiency of drug *in vivo*, animal models are used before implementing in clinical trials^45^. Anaphylaxis is the immediate allergic reaction initiated by the antigen-mediated crosslinking of IgE and FcεRI on mast cells. Immune pathways are involved in CD4^+^ T-cell T_H_2 development and antigen-specific IgE production through repeated antigen sensitisation^46^. In specific, OVA induction leads to the secretion of OVA-specific IgE by B cells, which is essential for the activation of mast cells, and rapid secretion of inflammatory mediators^27^. OVA challenges induce hypothermia and vasodilation by the increase of serum histamine levels^27^. The levels of histamine are associated with the severity of the anaphylaxis^47^. PCA is another well-defined animal model for local allergic reaction^48^. IgE sensitisation and antigen challenge lead to histamine release from mast cells and consequently produce local effects, such as increased vascular permeability, which results in tissue swelling and plasma extravasation. Evans blue dye binds to the extravasated plasma albumin and quantify the increased permeability^2^. According to our findings, oral administration of Avn C alleviated these reactions. Thus, we suggest that Avn C inhibited the allergic responses by suppressing the activation of mast cells.

Clinically, anti-histamine and corticosteroids have been used to treat various allergic diseases, although these drugs have side effects and are often ineffective^49^. Therefore, development of novel anti-allergic drugs is desirable. Our study showed the effects of Avn C from germinated oats against IgE-stimulated mast cell activation and mast cell-mediated systemic and local anaphylaxis. Oats are commonly accepted cereal grain in human diet. There is an epidemiological evidence that consumption of oats in the management of coronary heart disease and diabetes. Therefore, the regulatory agencies, Food and Drug Administration in USA and European Food Safety Authority verified the benefits of oats to human health^50^. Various research reported that germination improved the nutritional value and the increased Avns contents of oat^13,29^.

In conclusion, our results provided the beneficial properties of Avn C on mast cell-mediated allergic inflammation. Depending on these findings, we proposed that Avn C might be a promising candidate for the management of mast cell-mediated allergic diseases.

## Materials and Methods

### Reagents and cell culture

Anti-DNP IgE, DNP-HSA, *o*-phthaldialdehyde (OPA), 4-nitrophenyl N-acetyl-β-D-glucosamide, Dexa, and Histodenz™ were purchased from Sigma-Aldrich. RBL-2H3, RPMCs, and mBMMCs were cultured in Dulbecco’s Modified Eagle’s medium (DMEM), α-minimum essential medium (Gibco, Grand Island, NY), and Roswell Park Memorial Institute (RPMI)-1640 medium (Hyclone, Logan, UT) respectively, with 5% CO_2_ at 37 °C. To prepare complete media, 10% heat-inactivated fetal bovine serum (Gibco), 100 µg/mL streptomycin, 250 ng/mL amphotericin, and 100 U/mL penicillin G were added.

### Animals

Male Imprinting Control Region (ICR) mice (30–35 g, 6 weeks old) and Sprague-Dawley (SD) rats (240–280 g, 8 weeks old) were obtained from Dae-Han Experimental Animal Center (Deajeon, Korea). Throughout the study, animals (*n* = 5/cage) were provided with food and water *ad libitum* in a laminar air flow room maintained at 22 ± 2 °C with relative humidity of 55 ± 5% and 12 h light:dark cycles.

### Ethics statement

Animal care and treatment of were carried out in accordance with the guidelines of the Public Health Service Policy on the Humane Care and Use of Laboratory Animals. Animal experiments were approved by the Institutional Animal Care and Use Committee of Kyungpook National University (IRB # 2016-0001-123).

### Preparation of RPMCs

To isolate RPMCs, two SD rats were euthanized with CO_2_ and 40 mL Tyrode’s buffer were injected into the peritoneum. Then peritoneum was massaged gently for approximately 2 min. A small incision was made in the peritoneum, and then a solution containing peritoneal cells was aspirated using a Pasteur pipette. Cells were centrifuged at 150 × *g* for 2 min at room temperature, and the supernatant was removed and then resuspended with 1 mL phosphate buffered saline (PBS). RPMCs were separated from other peritoneal cells, such as macrophages and small lymphocytes, using 1 mL of 0.235 g/mL Histodenz™ solution, as previously described^48^. The purity and the viability of RPMCs were determined by toluidine blue (approximately 97%) and trypan blue (approximately 95%) staining.

### Preparation of mBMMCs

Mouse BMMCs were isolated from the femurs of male ICR mice (8 weeks old), as previously described^28^. Bone marrows were flushed using insulin syringe filled with 1 mL RPMI-1640 supplemented with 1 mM sodium pyruvate, 4 mM L-glutamine, 25 mM HEPES, MEM non-essential amino acids (Gibco), 50 µM 2-mercaptoethanol, 10 ng/mL IL-3, and 2 ng/mL stem cell factor (PeproTech EC, London, UK) into the cell culture dish (Sarstedt, Numbrecht, Germany) and then transferred into T flask (Life Sciences). Cells were cultured for 4 weeks and fluorescence-activated cell sorting (FACS) analysis was done to evaluate surface markers FcεRI and c-kit to confirm the mast cell purity. Cells expressing > 90% of FcεRI and c-kit were used for experiments (Supplementary Fig. S1).

### Cell viability

The viability of RBL-2H3, mBMMCs, and RPMCs were determined by MTT assay kit (Welgene, Seoul, Korea). Cells (3 × 10^4^ cells/well in a 96-well plate) were pre-treated with Avn C (0.01–100 µM) for 12 h, then 100 µL dimethyl sulfoxide was added to dissolve formed formazan crystals. The absorbance and viability of cells were detected and calculated, as described previously^31^.

### β-hexosaminidase release

RBL-2H3, mBMMCs, and RPMCs were sensitised with anti-DNP IgE (50 ng/mL), pre-treated with Avn C for 1 h and then challenged with DNP-HSA (100 ng/mL) for 2 h, 30 min, and 2 h, respectively. After incubation, the media was centrifuged at 150 × *g* for 5 min at 4 °C to separate cells. To measure the β-hexosaminidase release, 40 µL supernatant was transferred into 96-wells plate and incubated with 40 µL of a substrate solution for 1 h at 37 °C, as previously described^31^. The absorbance was detected at 405 nm using a spectrophotometer (Molecular Devices).

### Histamine release

RBL-2H3, mBMMCs, and RPMCs were sensitised with anti-DNP IgE (50 ng/mL), pre-treated with Avn C for 1 h and then challenged with DNP-HSA (100 ng/mL) for 4 h, 30 min, and 2 h respectively. Histamine levels in blood serum and release media were measured, as previously described^31^. To measure the fluorescence intensity, a fluorescence plate reader (Molecular Devices) was used at an excitation wavelength of 360 nm and an emission wavelength of 440 nm.

### Intracellular calcium

Fluo-3/AM (Invitrogen), a fluorescent indicator, was used to measure intracellular calcium content in RBL-2H3, mBMMCs, and RPMCs, as previously described^48^. To measure the fluorescent intensity a fluorescence plate reader was used at an excitation wavelength of 485 nm and an emission wavelength of 520 nm. The intracellular calcium levels were compared to those of untreated control cells to set 1 relative fluorescent unit.

### qPCR

RBL-2H3 were sensitised with anti-DNP IgE (50 ng/mL), pre-treated with Avn C for 1 h and then stimulated with DNP-HSA (100 ng/mL) for 1 h. Total cellular RNA was isolated, following the manufacturer’s protocol (RNAiso Plus kit, Takarabio, Shiga, Japan). Quantitative PCR was used to measure the mRNA expression of TNF-α, IL-4, and IL-6, as previously described^48^.

### ELISA

The levels of cytokines were measured by ELISA, as previously described^48^. RBL-2H3 were sensitised with anti-DNP IgE (50 ng/mL), pre-treated with Avn C for 1 h and then challenged with DNP-HSA (100 ng/mL) for 6 h. The assay was carried out using an ELISA kit (BD Biosciences) in a 96 well Nunc-Immune™ plate, following the manufacturer’s protocol. The substrate’s reaction was terminated, and then the absorbance was detected at a wavelength of 450 nm using a spectrophotometer.

### Western blot

Anti-DNP IgE sensitized RBL-2H3 were pre-treated with Avn C for 1 h, and then challenged with DNP-HSA for 7 min (Lyn and Syk), 15 min (PI3K and PLCγ1), 30 min (Akt), and 1 h (IκBα and NF-κB). Equal concentration of cellular protein was electrophoresed using 7.5–10% sodium dodecyl sulphate-polyacrylamide. The electrophoresed gel was transferred to a nitrocellulose membrane, as described previously^28^. Membrane was incubated with primary antibody (1:1000) in contrast to specific protein, and then with anti-IgG horseradish peroxidase-conjugated secondary antibody (1:2000). Immunodetection was performed using an enhanced chemiluminescence detection kit (Amersham, Piscataway, NJ).

### OVA-induced ASA

Each mouse (total *n* = 30, *n* = 5/group) was injected intraperitoneally with OVA mixture (100 μg OVA and 2 mg alum adjuvant**)** or PBS on days 0 and day 7, as previously described^48^. Subsequently, on days 9, 11, and 13, Avn C (0.1–10 mg/kg) and Dexa (10 mg/kg) were orally administered. On day 14, mice were challenged with intraperitoneal injection of 200 μg OVA, and then the rectal temperature was monitored and recorded in every 10 min intervals up to 90 min. Mice were euthanised after 90 min, and blood samples were collected from the abdominal artery.

### IgE-mediated PCA

Each mouse (total *n* = 30, *n* = 5/group) received intradermal injection of 0.5 µg/site anti-DNP IgE or PBS into ear to establish PCA reaction. Mice were monitored for 48 h. Then, Avn C (0.1–10 mg/kg) and Dexa (10 mg/kg) were administered per oral 1 h before DNP-HSA challenge. A mixture of DNP-HSA (1 mg/mouse) and 4% Evans blue (1:1) was intravenously injected into the tail vein and euthanised after 30 min. The ear thickness and absorbance were measured, as previously described^48^.

### Statistical analysis

All statistical data were analysed using SAS statistical software (SAS Institute, Cary, NC). The results are expressed as means ± SEM of three independent *in vitro* experiments and two independent *in vivo* experiments. Treatment effects were analysed using one-way analysis of variance followed by Tukey’s post hoc tests. *p* < 0.05 were considered as statistically significant.

## Supplementary information

Supplmentary file

## Data Availability

All the datasets generated and analysed during this study are included in this published article. The datasets generated during this study are available from the corresponding author on reasonable request.
