# Supplementary material for: Avenanthramide C from germinated oats exhibits anti-allergic inflammatory effects in mast cells
Source: Sci Rep. 2019 May 3;9:6884. doi: 10.1038/s41598-019-43412-2 (PMC6499795; doi:10.1038/s41598-019-43412-2)
Supplement: Supplementary file 1 — Supplmentary file [file 41598_2019_43412_MOESM1_ESM.docx]

**Avenanthramide C** **from germinated oats exhibits anti-allergic inflammatory effects in mast cells**

Hima Dhakal^1,2,^*, Eun-Ju Yang^3,^*, Soyoung Lee^4^, Min-Jong Kim^2^, Moon-Chang Baek^5^, Byungheon Lee^6^, Pil-Hoon Park^7^, Taeg Kyu Kwon^8^, Dongwoo Khang^9,†^, Kyung-Sik Song^3,10,†^, Sang-Hyun Kim^1,2,†^

^1^Cell & Matrix Research Institute, ^2^Department of Pharmacology, ^5^Department of Molecular Medicine, ^6^Department of Biochemistry and Cell Biology, School of Medicine, Kyungpook National University, Daegu, Republic of Korea

^3^Research Institute of Pharmaceutical Sciences, College of Pharmacy, Kyungpook National University, Daegu, Republic of Korea

^4^Immunoregulatory Materials Research Center, Korea Research Institute of Bioscience and Biotechnology, Jeongeup, Republic of Korea

^7^College of Pharmacy, Yeungnam University, Gyeongsan, Republic of Korea

^8^Department of Immunology, School of Medicine, Keimyung University, Daegu, Republic of Korea

^9^Department of Physiology, School of Medicine, Gachon University, Incheon, Republic of Korea

^10^GHAM BioPharm Co. Ltd., College of Pharmacy, Kyungpook National University, Daegu, Republic of Korea

*Contributed equally to this work

^†^Correspondence and requests for materials should be addressed to shkim72@knu.ac.kr (S.H.K.), kssong@knu.ac.kr (K.S.S.) or dkhang@gachon.ac.kr (D.K.)

**Supplementary Method 1.**

**Extraction, isolation, and identification of Avn C (Figure 1A)**

Daeyang oat powder (500 g; germinated for 64 h; harvested in 2016) was provided from National Institute of Crop Science (Rural Development Administration, Wanju-Gun, Republic of Korea). To remove fat, oat powder was treated with *n*-hexane (2 L) and kept for 7 days at room temperature, and then decanted to obtain the residue. The defatted residue was extracted with 80 % ethanol (EtOH, 1 L) at 50 °C for 30 min three times and filtered using a filter paper and Büchner funnel. To prepare Avn C rich fraction, filtrate was concentrated by a rotary evaporator (EYELA, Tokyo, Japan) to yield an oily 80 % EtOH-soluble fraction (59.9 g), suspended in 200 mL methanol (MeOH) for 10 min, and then centrifugation was done at 3000 rpm for 10 min at 20 °C. This fraction was subjected to silica gel column chromatography (230 ~ 400 mesh; Ø 7.5×35.0 cm; chloroform:MeOH:H_2_O = 10:7:1) to yield 3 fractions (Fr. 1 ~ 3). Fr. 2 (1.1 g) was re-chromatographed with an ODS column (150 μm; Ø 2.8×35.0 cm; 40 % to 100 % MeOH) to obtain 4 fractions (Fr. 2-1 ~ Fr. 2-4). Fr. 2-2 (97 mg) was applied to an HPLC (Luna 5 μ C18(2), Phenomenex, Torrance, CA) and eluted with the mobile phases composed of H_2_O (solvent A) and MeOH (solvent B) (Burdick & Jackson, Muskegon, MI), each containing 0.1 % acetic acid (Wako, Tokyo, Japan). Gradient conditions were carried out as follows; start with 40 % of solvent B and held for 45 min, then the solvent B was increased to 100 % in 5 min (total run time = 50 min), a flow rate 3.0 mL/min run rate, and detection condition at 254 nm using a UV detector (DIONEX Ultimate, Thermo Scientific, Germering, Germany). A major peak (compound **1**) appeared at 42.5 min, compound **1** was collected and evaporated. The purity of Avn C was ˃ 95 % as determined by HPLC analysis (Eun-Yeong Yun, The anti-inflammatory effects of avenanthramide C from the germinated oat seeds in BV-2 cells. 2018. MS thesis. Department of Pharmacy, Graduate School, Kyungpook National University, pp 17-25). Using NMR analysis (Bruker Avance Digital 500 NMR spectrometer, Karlsruhe, Germany) of compound **1** (21 mg) was identified as Avn C (gift from GHAM BioPharm Co. Ltd, Daegu, Republic of Korea) by comparing its NMR data with those in literature and of authentic Avn C^1^. The NMR data were expressed as chemical shifts (δ, ppm) relative to an internal standard, tetramethylsilane (TMS).

Avn C (1). ^1^H-NMR (500 MHz, MeOH-*d*_4_): δ 8.45 (1H, d, *J*=9.0 Hz, H-3), 7.52 (1H, d, *J*=3.2 Hz, H-6), 7.5 (1H, d, *J*=15.6 Hz, H-7'), 7.07 (1H, d, *J*=1.7 Hz, H-2'), 7.02 (1H, dd, *J*=9 and 3.2 Hz, H-4), 6.97 (1H, dd, *J*=8.2 and 1.7 Hz, H-6'), 6.79 (1H, d, *J*=8.2 Hz, H-5'), 6.46 (1H, d, *J*=15.6 Hz, H-8').

**Supplementary Method 2.**

**FACS analysis of mBMMCs.**

The harvested mBMMCs were washed once with phosphate buffer saline (PBS) and then cells (5 × 10^5^/well) were incubated with the fluorescence labelled antibodies for 30 min at 4 °C. Anti-mouse IgG-PE and anti-mouse IgG-FITC were purchased from Santa Cruz Biotechnology (Paso Robles, CA). Anti-mouse FcεRI-PE, CD117-PE, and FcεRI-FITC were purchased from Invitrogen (Carlsbad, CA), BD Biosciences (San Diego, CA), and Biolegend (San Diego, CA) respectively. After incubation, cells were centrifuged at 150 *g* for 30 s, supernatant removed, washed once with PBS, and then analysed using FACS Calibur flow cytometer (BD Biosciences) and Cell Quest Pro software (S1).

**Supplementary Figure S1.** Representative histogram of FACS analysis.


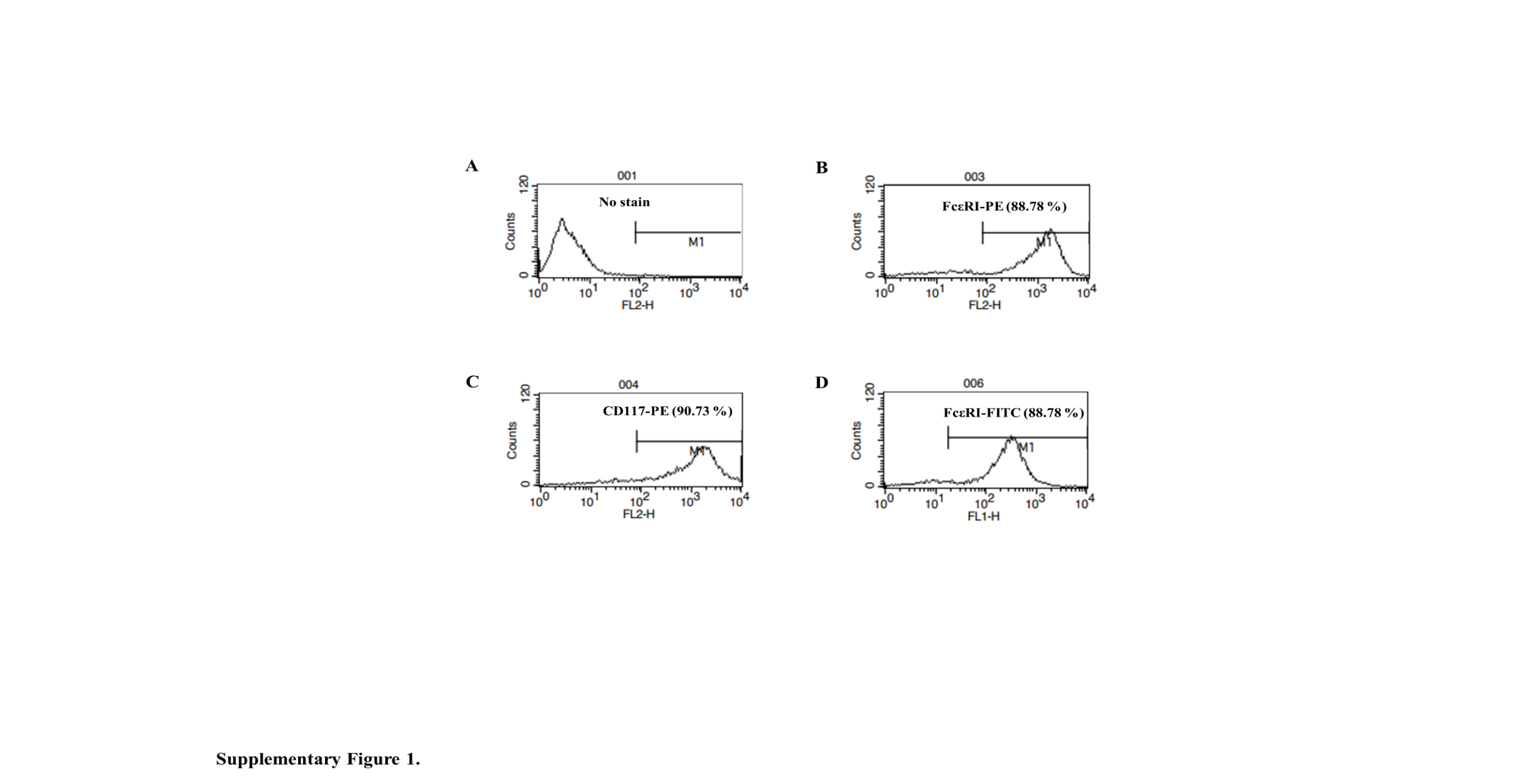


(A) No stained cells. The cells were stained with (B) FcεRI-PE, (C) CD117-PE. (D) FcεRI-FITC. Numbers shown in the histogram represent the percentage of positive cells. Data reflect a single representative experiment.

**Supplementary Figure S2.**

**Full-length Western blot corresponding to Figure 2E.**


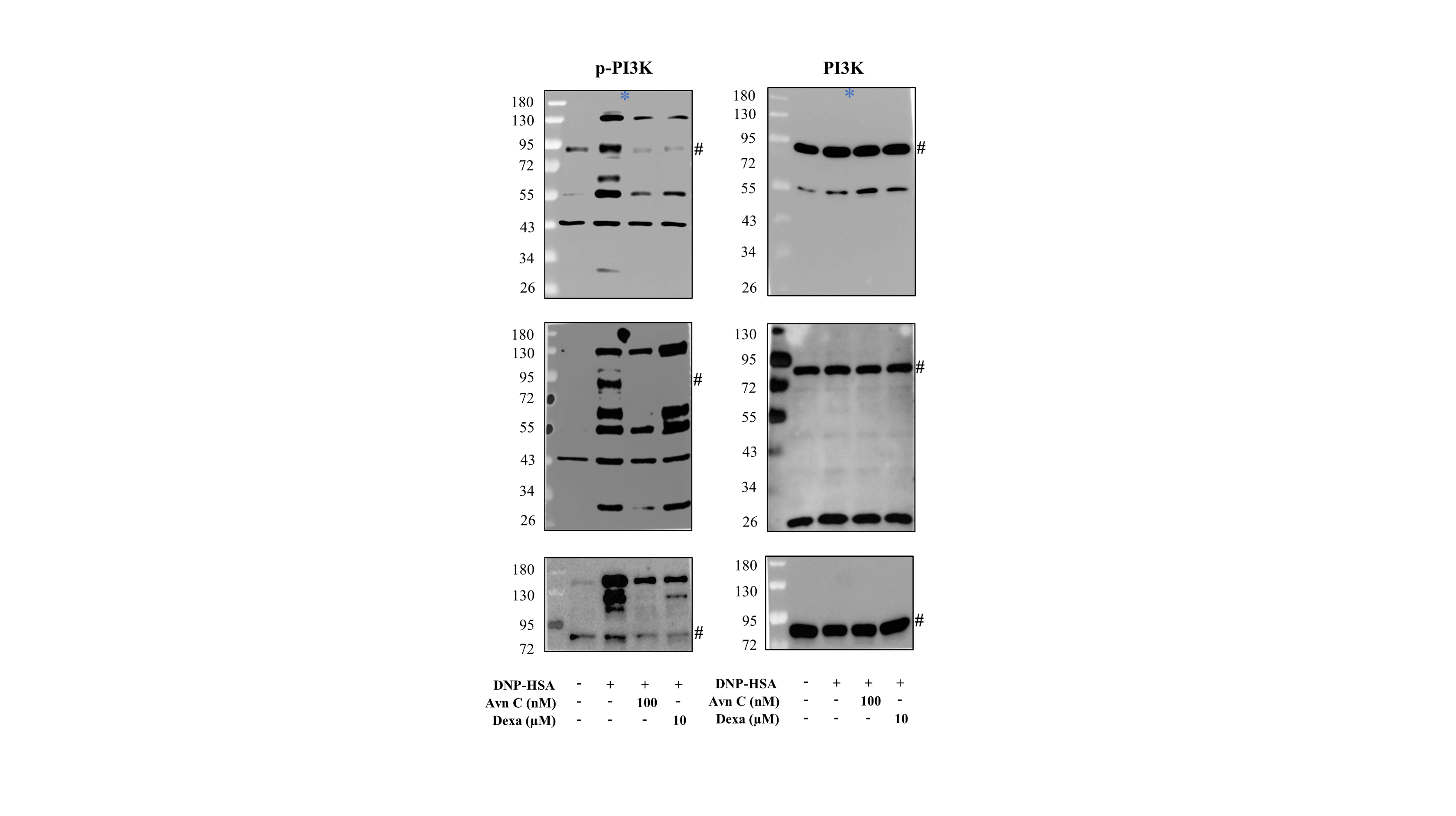


Supplementary information of Figure 2E: phospho-PI3K (85 kDa) and total PI3K (85 kDa). Hash (#) denotes target band. Blots with asterisk (blue) were used in main Figure.


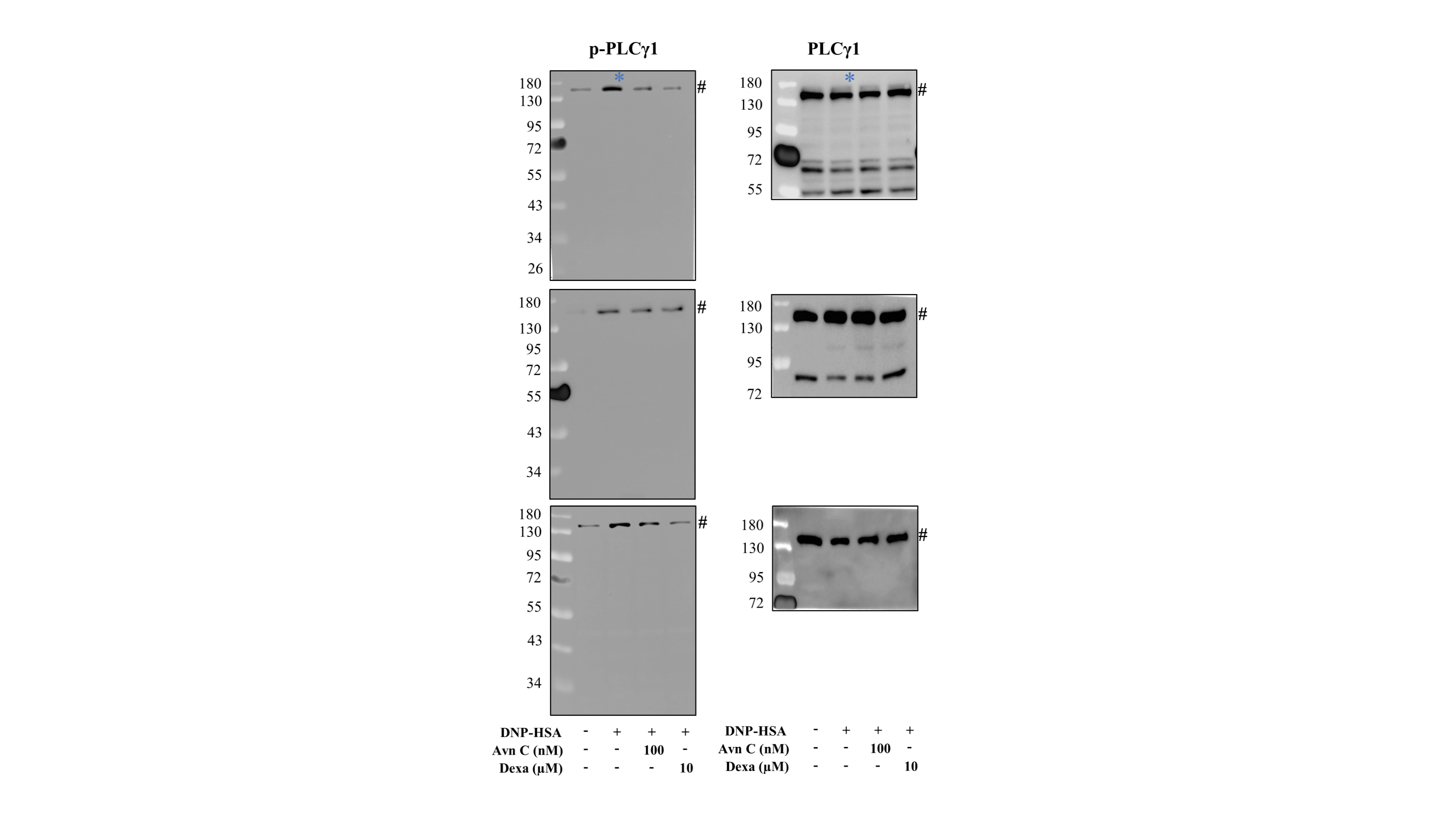


Supplementary information of Figure 2E: phospho-PLCγ1 (140 kDa) and total PLCγ1 (140 kDa). Hash (#) denotes target band. Blots with asterisk (blue) were used in main Figure.

**Full-length Western blot corresponding to Figure 4.**


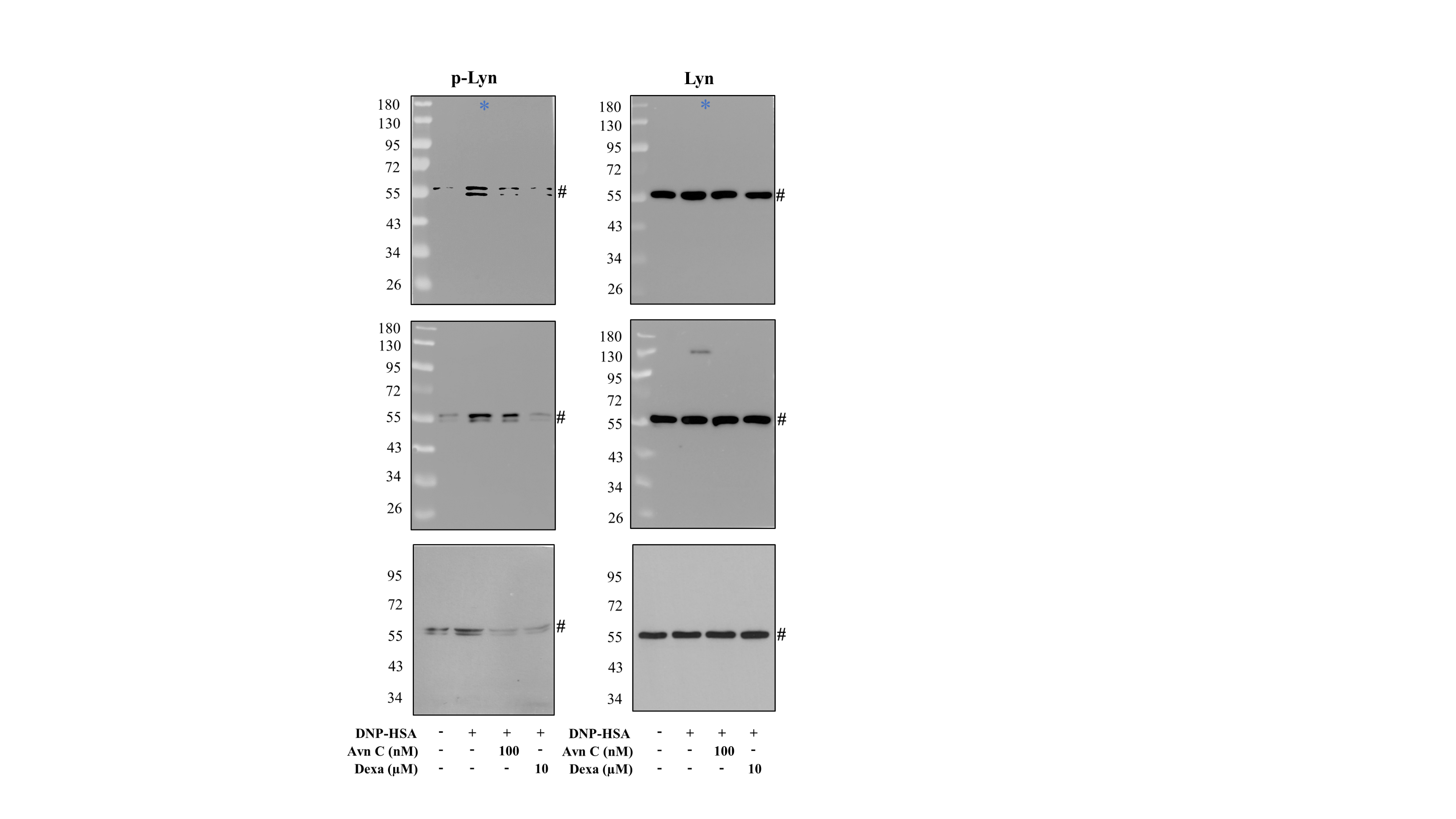


Supplementary information of Figure 4: phospho-Lyn (56 kDa) and total Lyn (56 kDa). Hash (#) denotes target band. Blots with asterisk (blue) were used in main Figure.


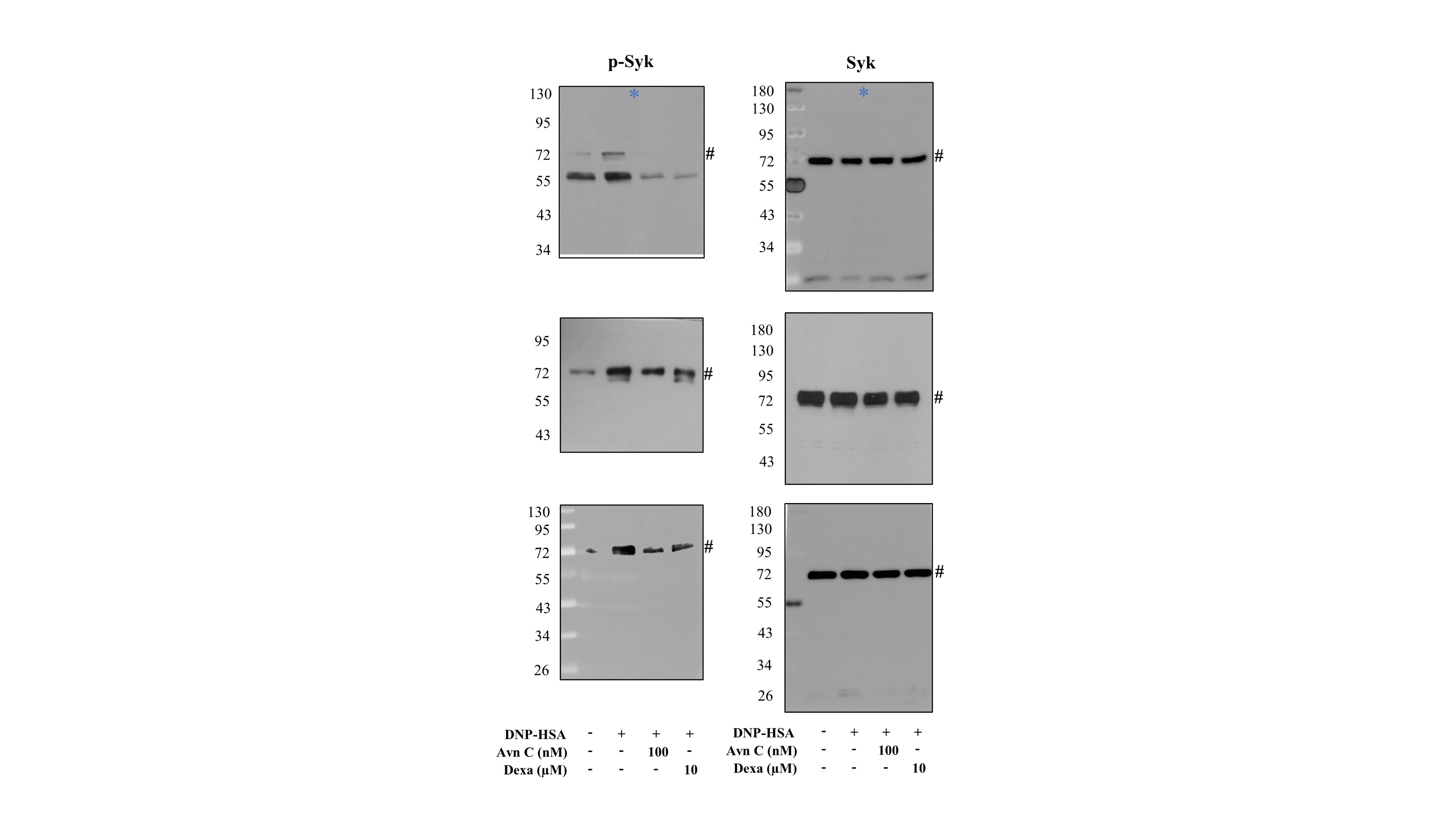


Supplementary information of Figure 4: phospho-Syk (72 kDa) and total Syk (72 kDa). Hash (#) denotes target band. Blots with asterisk (blue) were used in main Figure.


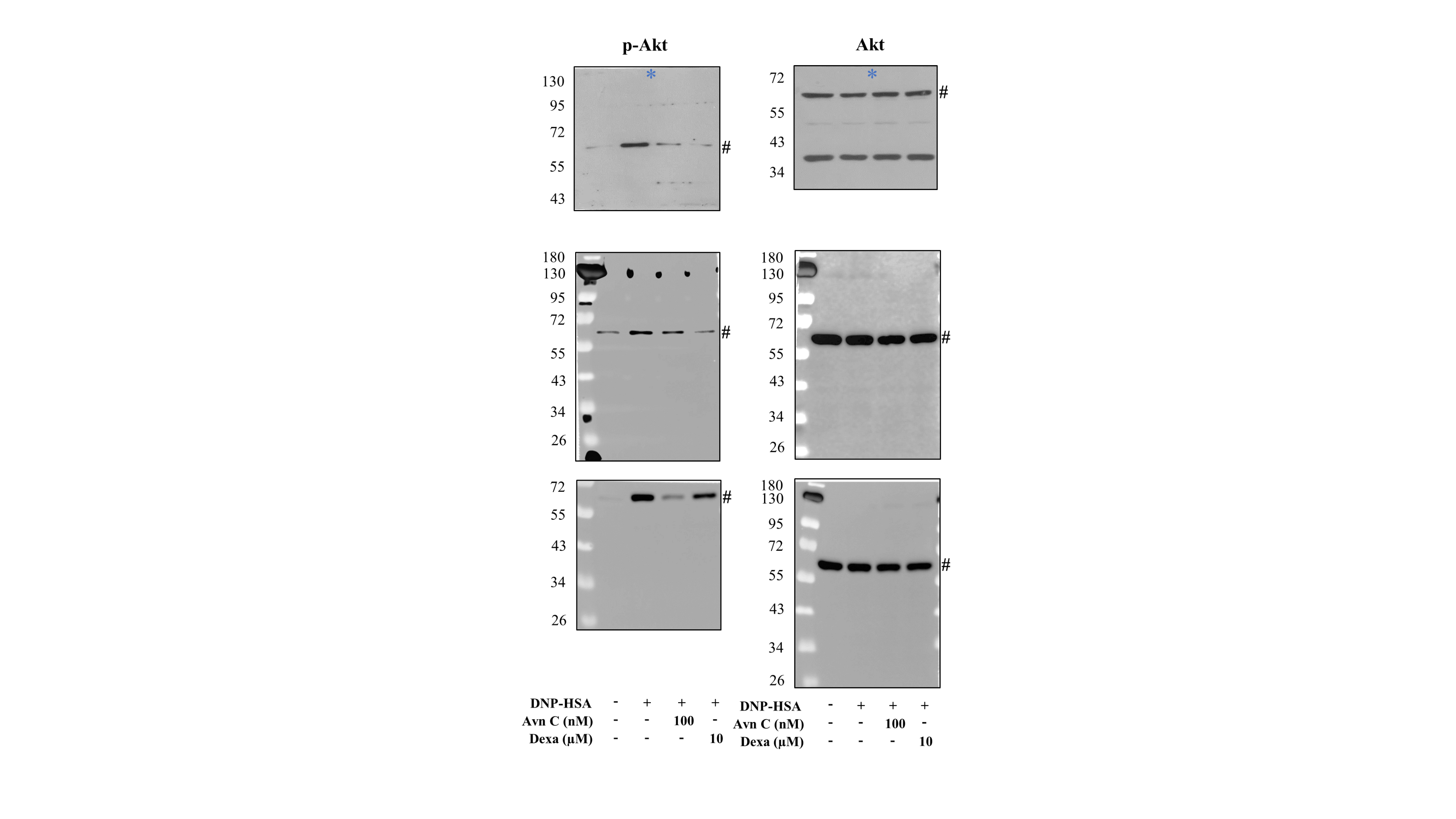


Supplementary information of Figure 4: phospho-Akt (60 kDa) and total Akt (60 kDa). Hash (#) denotes target band. Blots with asterisk (blue) were used in main Figure.


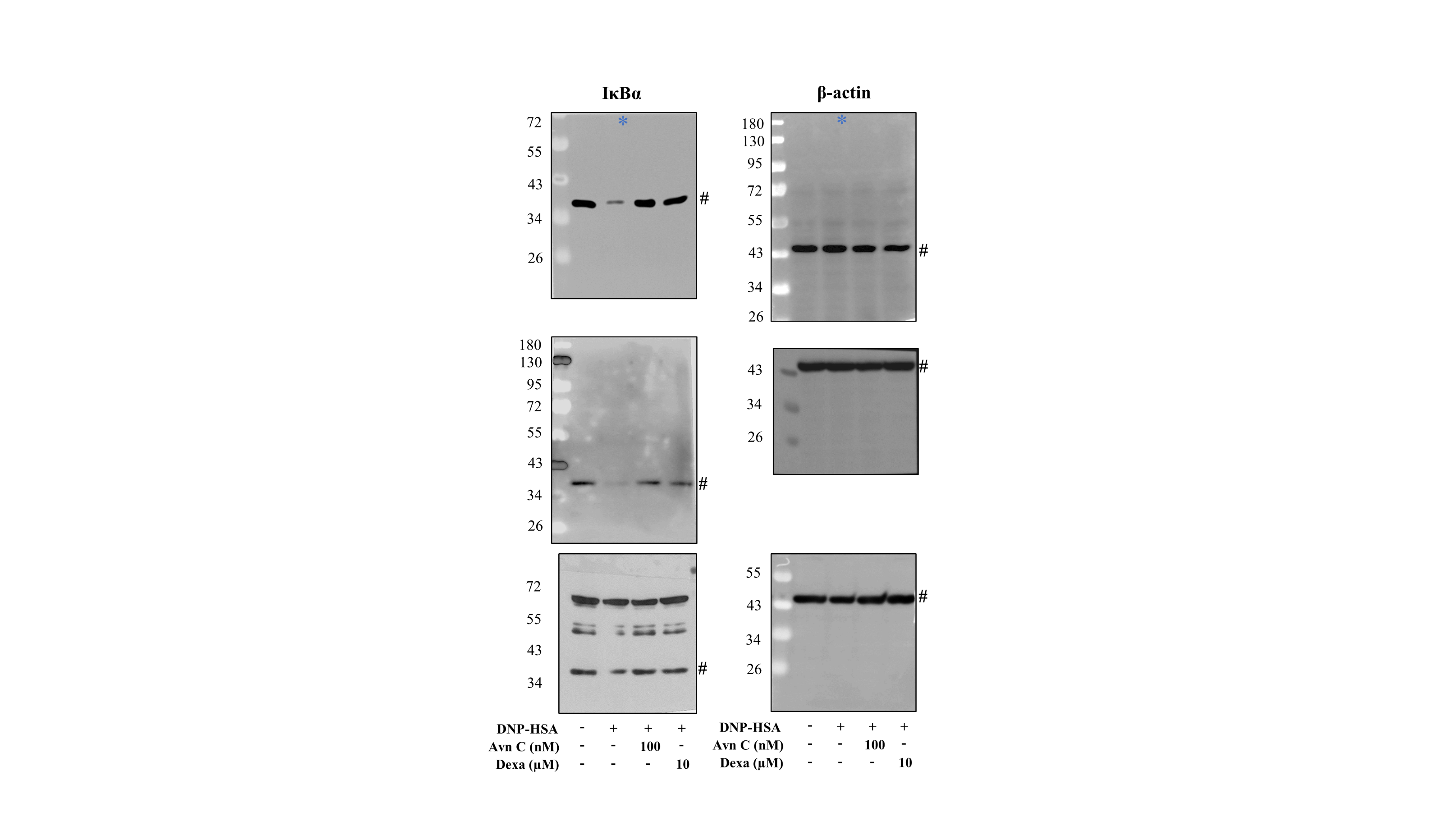


Supplementary information of Figure 4. Full length blot of IκBα **(**37 kDa) and β-actin (43 kDa). Hash (#) denotes target band. Blots with asterisk (blue) were used in main Figure.


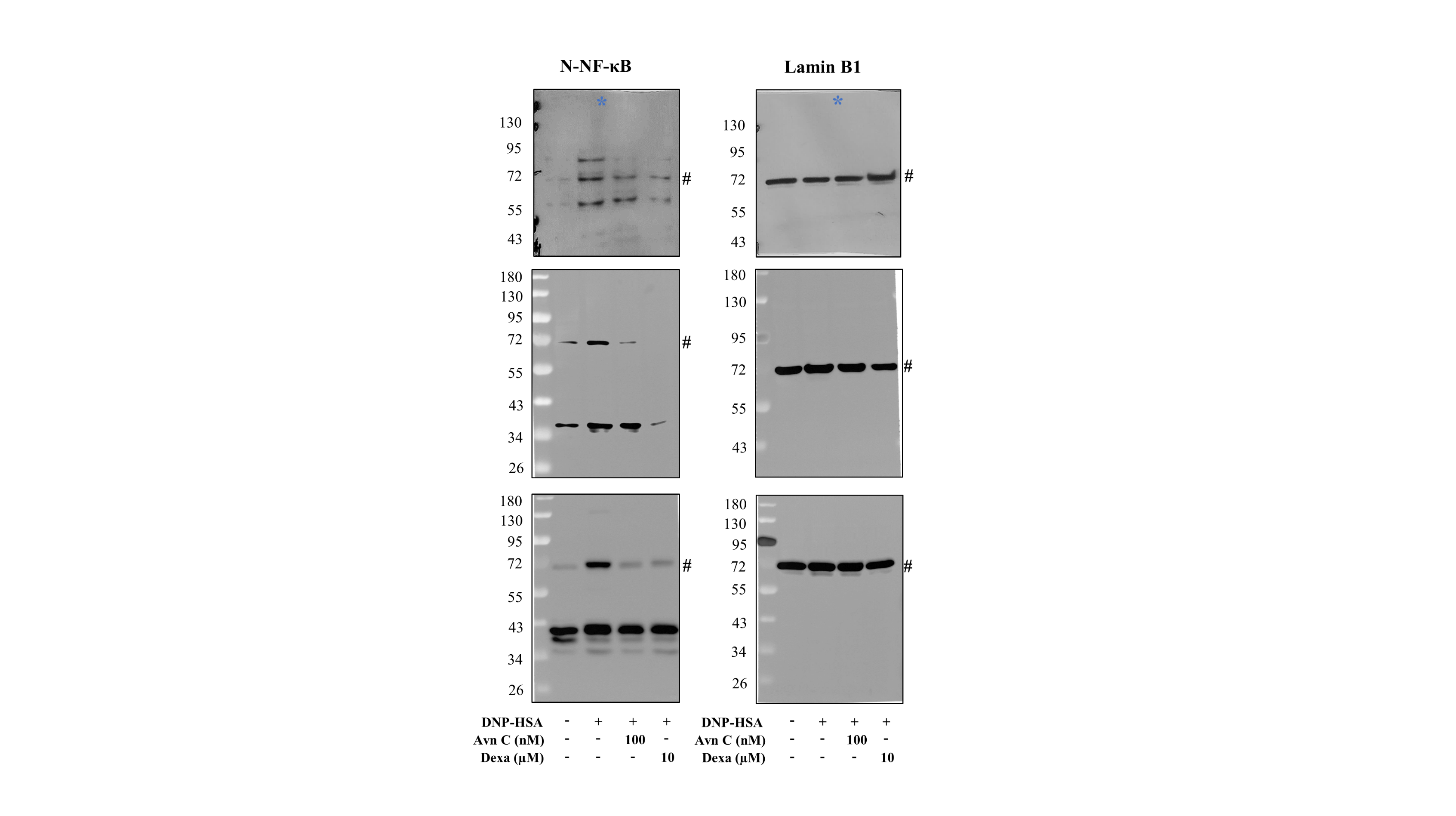


Supplementary information of Figure 4. Full length blot of nucleus NF-κB (65 kDa) and lamin B1 (67 kDa). Hash (#) denotes target band. Blots with asterisk (blue) were used in main Figure.

**Antibodies used in the manuscript**

1. NF-κB (Santa Cruz, sc-109, rabbit polyclonal, 1:1000)
2. IκBα (Santa Cruz, sc-371, rabbit polyclonal, 1:1000)
3. Lamin B1 (Santa Cruz, sc-6217, goat polyclonal, 1:1000)
4. β-actin (Santa Cruz, sc-8432, mouse monoclonal, 1:1000)
5. Phospho-Lyn (Cell Signaling Technology, #2731, rabbit polyclonal, 1:1000)
6. Phospho-Syk (Cell Signaling Technology #2711, rabbit polyclonal, 1:1000)
7. Phospho-Akt (Cell Signaling Technology, #9271, rabbit polyclonal, 1:1000)
8. Phospho-PI3K (Cell Signaling Technology, #4228, rabbit polyclonal, 1:1000)
9. Phospho-PLCγ (Cell Signaling Technology, #2821, rabbit polyclonal, 1:1000)
10. Lyn (Cell Signaling Technology, #2732, rabbit polyclonal, 1:1000)
11. Syk (Cell Signaling Technology, #2712, rabbit polyclonal, 1:1000)
12. Akt (Cell Signaling Technology, #9272, rabbit polyclonal, 1:1000)
13. PI3K (Cell Signaling Technology, #4292, rabbit polyclonal, 1:1000)
14. PLCγ (Cell Signaling Technology, #2822, rabbit polyclonal, 1:1000)
15. IgG, PE (Santa Cruz, sc-3798, anti-mouse, 1:100)
16. IgG, FITC (Santa Cruz, sc-3699, anti-mouse, 1:100)
17. FcεRI, PE (Invitrogen, Lot-7068634, anti-mouse, 1:100)
18. CD117, PE (BD Biosciences, Lot-7068634, anti-mouse, 1:100)
19. FITC (Biolegend, Lot-B211394, anti-mouse, 1:100)

**References**

1 Wise, M. L. Effect of chemical systemic acquired resistance elicitors on avenanthramide biosynthesis in oat (Avena sativa). *J Agric Food Chem.* **59**, 7028-7038, doi:10.1021/jf2008869 (2011).
